# Feasibility of a meditation intervention for stroke survivors and informal caregivers: a randomized controlled trial

**DOI:** 10.1186/s40359-022-01031-z

**Published:** 2023-01-12

**Authors:** Jennifer E. S. Beauchamp, Anjail Sharrief, Alejandro Chaoul, Tahani Casameni Montiel, Mary F. Love, Stanley Cron, Alan Prossin, Sudhakar Selvaraj, Deniz Dishman, Sean I. Savitz

**Affiliations:** 1 Cizik School of Nursing Department of Research and the Institute for Stroke and Cerebrovascular Disease, University of Texas Health Science Center at Houston, 6901 Bertner Avenue, Suite 580D, Houston, TX 77030 USA; 2 McGovern Medical School Department of Neurology and the Institute for Stroke and Cerebrovascular Disease, The University of Texas Health Science Center at Houston, 6410 Fannin St, Suite 1014, Houston, TX 77030 USA; 3 Mind Body Spirit Institute, The Jung Center of Houston, 5200 Montrose Ave., Houston, TX 77006 USA; 4 Cizik School of Nursing Department of Research, The University of Texas Health Science Center at Houston, 6901 Bertner Avenue, Suite 582, Houston, TX 77030 USA; 5 College of Nursing, University of Houston, 14000 University Boulevard, #367G, Sugar Land, Houston, TX 77479 USA; 6 Cizik School of Nursing, University of Texas Health Science Center at Houston, 6901 Bertner Avenue, Suite SON 561, Houston, TX 77030 USA; 7 McGovern Medical School Department of Psychiatry and Behavioral Sciences, The University of Texas Health Science Center at Houston, 1941 East Road, Suite BBS 2310, Houston, TX 77054 USA; 8 Louis Faillace Department of Psychiatry and Behavioral Science, McGovern Medical School, The University of Texas Health Science Center at Houston, 1941 East Road, Suite BBS 3152, Houston, TX 77054 USA; 9 Cizik School of Nursing Department of Research, The University of Texas Health Science Center at Houston, 6901 Bertner Avenue, Suite SON580C, Houston, TX 77030 USA; 10 McGovern Medical School Department of Neurology and the Institute for Stroke and Cerebrovascular Disease, The University of Texas Health Science Center at Houston, 6431 Fannin, Suite MSB-7.128, Houston, TX 77030-1503 USA

**Keywords:** Stroke, Depression, Meditation, Mental health

## Abstract

**Background:**

Depressive symptoms are a significant psychological complication of stroke, impacting both survivors and informal caregivers of survivors. Randomized controlled trials are needed to determine optimal non-pharmacological strategies to prevent or ameliorate depressive symptoms in stroke survivors and their informal caregivers.

**Methods:**

A prospective, randomized, parallel-group, single-center, feasibility study. Participants were assigned to a 4-week meditation intervention or expressive writing control group. The intervention comprised four facilitator-led group meditation sessions, one session per week and building upon prior session(s). Descriptive statistics were used to examine the proportion of eligible individuals who enrolled, retention and adherence rates, and the proportion of questionnaires completed. Data were collected at baseline, immediately after the 4-week intervention period, and 4 and 8 weeks after the intervention period. Secondary analysis tested for changes in symptoms of depression (Center for Epidemiologic Studies-Depression [CES-D]), anxiety [State-Trait Anxiety Inventory for Adults (STAI)], and pain (Brief Pain Inventory-Short Form) in the intervention group via paired *t* tests. Linear mixed models were used to compare longitudinal changes in the measures between the groups. Intervention and trial design acceptability were preliminary explored.

**Results:**

Seventy-one (77%) individuals enrolled and 26 (37%) completed the study (baseline and 8-week post-intervention visits completed). Forty-two (66%) participants completed baseline and immediate post-intervention visits. Mean questionnaire completion rate was 95%. The median meditation group session attendance rate for the intervention group was 75.0%, and the mean attendance rate was 55%. Non-significant reductions in CES-D scores were found. Paired *t* tests for stroke survivors indicated a significant reduction from baseline through week 8 in BPI-sf severity scores (*p* = 0.0270). Repeated measures analysis with linear mixed models for informal caregivers indicated a significant reduction in in STAI-Trait scores (F [3,16.2] = 3.28, *p* = 0.0479) and paired *t* test showed a significant reduction from baseline to week 4 in STAI-Trait scores (mean = − 9.1250, 95% CI [− 16.8060 to 1.4440], *p* = 0.0262). No between-group differences were found.

**Conclusions:**

Future trials will require strategies to optimize retention and adherence before definitive efficacy testing of the meditation intervention.

*Trial registration*: ClinicalTrials.gov Identifier: NCT03239132. Registration date: 03/08/2017

**Supplementary Information:**

The online version contains supplementary material available at 10.1186/s40359-022-01031-z.

## Background

Stroke is a leading cause of morbidity and disability [1]. Annual stroke occurrence and years of life lost due to acute and chronic disabilities have increased [1]. Greater emphasis on stroke recovery could yield both clinical and societal benefits. Depressive symptoms are reported in approximately one-third of stroke survivors (SS) and are associated with comorbid anxiety and pain, and informal caregiver (IC; e.g., spouse) psychological problems [2–4]. The neurologic deficits and protracted inflammatory response persisting beyond the acute phase of stroke may facilitate the development, severity, and prolongation of depressive symptoms beyond that seen with other acute medical events [5]. Evidence-based non-pharmacological treatment strategies (e.g., cognitive behavioral therapy and mindfulness meditation), often in combination with pharmacotherapy, for post-stroke depression, anxiety, and pain exist [6–8]. However, the quality of the existing studies is limited (e.g., small samples, lack of comparator groups and longitudinal data), and optimal treatment strategies (e.g., timing of the intervention) are unknown [9].

Breathing-based interventions (e.g., meditation) have been shown to reduce depressive symptoms in cancer populations [10]. Such investigations are largely absent in the stroke population [6]. Recommendations exist for intervention studies that include SS and IC with a focus on the health and well-being of both members of the dyad [11, 12]. Our primary aim was to determine the feasibility of a randomized controlled trial (RCT) of a 4-week meditation intervention in SS and IC. Our secondary aim was to investigate the effect of the intervention on symptoms of depression, anxiety, and pain. We hypothesized that a meditation intervention leads to decreases in depression, anxiety, and pain symptoms. We preliminarily explored the acceptability of the intervention and trial design.

## Methods

### Participants

Due to funding restraints, we aimed to enroll 24 SS-IC dyads into the intervention group and 12 dyads into the control group. Individuals were eligible if a dyad was not possible. Potential participants were approached during an outpatient stroke clinic visit. SS are seen in our stroke clinic approximately 2–6 weeks post-hospitalization, and visits are repeated at 3, 6, and 12 months. SS were included if they were at least 18 years old, could speak and read English, could provide written informed consent, had an ischemic stroke, intracerebral hemorrhagic stroke, or transient ischemic attack (recurrent or first-time stroke event) within the past 12 months, and currently lived at home. SS motivation and readiness to participate in recovery has been shown to be present outside of the immediate acute stroke period [13]. IC were included if they were at least 18 years old, could speak and read English, could provide written informed consent and self-identified as an IC of a SS. To reflect standard practice, SS and IC were eligible to participate regardless of whether they were currently receiving anti-depressant or anxiolytic medications. SS residing outside of the home (e.g., extended rehabilitation facility) were excluded to assist us in limiting the enrollment of survivors with major cognitive and functional disabilities and impairments that may limit their ability to participate. SS and IC were excluded for psychosis, illegal substance use, suicidal ideation, or self-report current engagement in psychotherapy or meditation practices of any kind. A brief Montreal Cognitive Assessment (MoCA) [14] score of < 9 was used to determine the absence of severe cognitive impairment in assessing SS capability of providing consent. However, it is important to recognize that the MoCA may not fully capture one’s ability to understand key informed consent elements and provide informed consent. If a SS had a brief MoCA score < 9, but appears to understand key informed consent elements (e.g., potential risks) demonstrating adequate consent capacity, written informed consent was obtained. Written informed consent was obtained from all participants prior to enrollment. Approval prior to study onset was obtained from the university’s Institutional Review Board, and all procedures were followed in accordance with guidelines. All research was conducted at a medical center in Southeastern Texas.

### Sample size

The estimate of effect size was based on a study that examined the feasibility and efficacy of meditation to improve depressive symptoms as a secondary outcome measure [15]. Based on that study’s effect size (d = 0.65), a repeated measures analysis for change in instrument score only within the intervention group would have 80% power when the sample size is n = 21. Considering an attrition rate of 12%, the targeted enrollment was 36 dyads. We estimated our attrition rate based on our experiences successfully completing other low-risk clinical trials. Power calculations were performed with G*Power 3.1.

### Randomization

Using a two-group blocked randomization scheme with a 2:1 allocation, dyads or individual participants were assigned to the intervention or control groups in blocks of six. The statistician generated the random allocation sequence in SAS software and was not involved in enrollment or intervention assignment. The randomized list was uploaded to Research Electronic Data Capture (REDCap) [16] where it was used to provide the group assignment.

### Intervention group

The breathing-based meditation intervention occurred in 4-week blocks and comprised four group sessions, one session per week, conducted by a senior meditation teacher with 20 years of experience in mind–body interventions (A.C.) [17]. The meditation group sessions were held at a nursing school in the same medical center as the recruitment site. Each session lasted approximately 1 h and built upon the previous session(s) to allow for repetitive practice. Participants were given pre-recorded audio and written instructions of the first three sessions at the end of each corresponding session and encouraged to practice on their own on days when they did not meet with the meditation teacher and daily thereafter. Participants were asked to document home practices using a daily meditation practice questionnaire. Daily meditation practice adherence was assessed as the proportion of daily meditation practice questionnaires that indicated the participant had meditated.

Session one focused on learning breathing techniques to calm one’s mind. Session two added learning how to still one’s body, silence one’s speech, and open one’s mind. Session three added learning to stay deeper in one’s connection through mindfully maintaining and deepening the connection through breathing with more openness and awareness and connecting to one’s qualities of warmth. Session four included expanding the warmth of one’s heart with loving kindness for others without forgetting oneself and an overview of the previous sessions, practice of the entire program, and an opportunity to ask questions. The meditation intervention was developed from the Tibetan tradition of the three doors which includes the body, speech, and mind [18]. A fidelity checklist was employed to evaluate the provision of the sessions by the specialist. All meditation intervention sessions were audio recorded, and every tenth session was reviewed by a member of the research study not involved in the intervention sessions for intervention fidelity by the senior meditation teacher.

### Control group

Participants assigned to the control group received four educational handouts on an expressive writing technique via email or mail at times corresponding to each of the four sessions in the intervention group. Handout one highlighted the benefits of writing about life experiences. Handout two focused on exploring emotions and thoughts about traumatic experiences through writing. Handout three outlined ideas for developing a writing practice. Participants were asked to write about anything of personal relevance or importance to them. Handout four focused on writing about experiences and expressing emotions through writing. Additional prompts to engage in writing techniques were not provided. A control group allowed for a realistic examination of enrollment and retention rates and randomization procedures. 

### Measures

#### Questionnaires

Participants were formally assessed at four time-points: pre-intervention or baseline and post-intervention (0 [immediately], 4, and 8 weeks after intervention completion) using REDCap electronic questionnaires [16, 19]. Baseline demographic data were obtained. The Center for Epidemiologic Studies Depression (CES-D) scale was used to assess depressive symptom severity with a cutoff score of 16 or higher to identify risk for clinical depression and has been validated in community-dwelling adults with stroke [20, 21]. Diagnostic Interview for Genetic Studies (DIGS) [22] questionnaires were administered via telephone by the PI or a trained research staff member at baseline. The DIGS [22] questionnaires were reviewed by a psychiatrist (A.P.) and the PI (JB), using algorithms to determine the presence or history of psychiatric disorders and conditions. Anxiety was measured using the State-Trait Anxiety Inventory for Adults (STAI) [23]. Range of scores for each State and Trait subscale is 20–80, with higher scores indicating greater levels of anxiety. A cutoff score of 40 on the State-Anxiety subscale is routinely used to indicate clinical levels of anxiety [23]. The Brief Pain Inventory-Short Form (BPI-sf) was used to assess pain intensity on a 0 to 10 scale with 0 indicating “no pain” and 10 indicating “pain as bad as you can imagine” and interference on a 0 to 10 scale with 0 indicating “does not interfere” and 10 indicating “completely interferes” in the past 24 h [24]. While there are no published reports of the sensitivity and specificity of the STAI or BPI-sf in screening for post-stroke anxiety or pain, our experience is that these instruments are commonly used with the post-stroke community [25, 26]. Participants assigned to the intervention group completed daily meditation practice questionnaires during the 4-week intervention and 8-week post-intervention phases. The questionnaire included five questions evaluating the daily total time in minutes and frequency of the day’s practice as number of times per day, whether the practice was alone as a yes or no response, and how the participant felt (e.g., happy, anxious, sad, or depressed) before and after the practice. An open-ended question was posed to obtain any additional thoughts, notes, or comments about the practice.

#### Blood samples

In order to understand the feasibility of obtaining and storing blood samples over time, samples were collected at the four data collection time-points. Samples were centrifuged to obtain 500 µl plasma aliquots and stored at − 80 °C for future analysis of biomarkers (e.g., pro-inflammatory cytokines). Research of the effects of mind–body interventions suggests multiple biological pathways (e.g., inflammatory and neuroendocrine) that may benefit SS [27].

#### Adherence to the intervention protocol

Intervention fidelity strategies were employed, including a fidelity checklist. All in-person meditation intervention sessions were recorded, and every tenth session was reviewed for intervention fidelity. Daily participant meditation practice adherence was assessed as the proportion of daily meditation practice questionnaires that indicated the participant had meditated.

#### Acceptability of the intervention and trial design

Immediately after each final (fourth) meditation session, the PI and senior meditation teacher informally interviewed participants assigned to the intervention group to assess the acceptability of the intervention and trial design. These unstructured group interviews were audio recorded and manually transcribed by a member of the research team. Reasons for non-participation were obtained from eligible individuals who declined to participate [16, 19]. Participant field notes were documented in REDCap [16, 19] by the PI and research staff.

### Analysis

Participant scores for the instruments were calculated as the mean of the answered items multiplied by the total number of items in the scale (or just the mean of the answered items for the BPI-sf). This method addresses missing data by using the scale items that were answered by a participant to calculate their scale score [28]. Descriptive statistics in the form of frequencies and percentages were used to examine demographic and clinical characteristics. Based on our experiences completing other low-risk, longitudinal RCTs with community-dwelling SS, pre-determined criteria for determining the success of the feasibility study were based on 80% or more of eligible individuals enrolling. Additionally, 80% or more needed to complete at least one questionnaire at baseline and one questionnaire at 8 weeks post-intervention. Participants needed to complete 75% or more of the questionnaires during the visits. Intervention participants needed to attend 75% or more of meditation sessions.

Quantitative data analysis was based on intention-to-treat principle. The analysis tested for changes in CES-D, STAI, and BPI-sf scores for the intervention group only. These within-group comparisons between time points were conducted with paired *t* tests. Although the study had adequate statistical power to analyze change only within the intervention group, additional analyses were conducted using repeated measures analysis with linear mixed models to compare the intervention and control groups for changes over time in the CES-D, STAI and BPI-sf questionnaires. Separate models were conducted for the SS and IC participants as individuals and not as dyads. Qualitative data analyses included two independent members of the research team reviewing the transcriptions of the unstructured group interviews and the field notes and grouping the information into categories. As a group, a third member of the team worked with the independent members to review and determine the final categories. Analysis included reviewing the reasons for declining study participation and grouping the reasons into categories.

## Results

### Characteristics of the sample

**Fig. 1:**
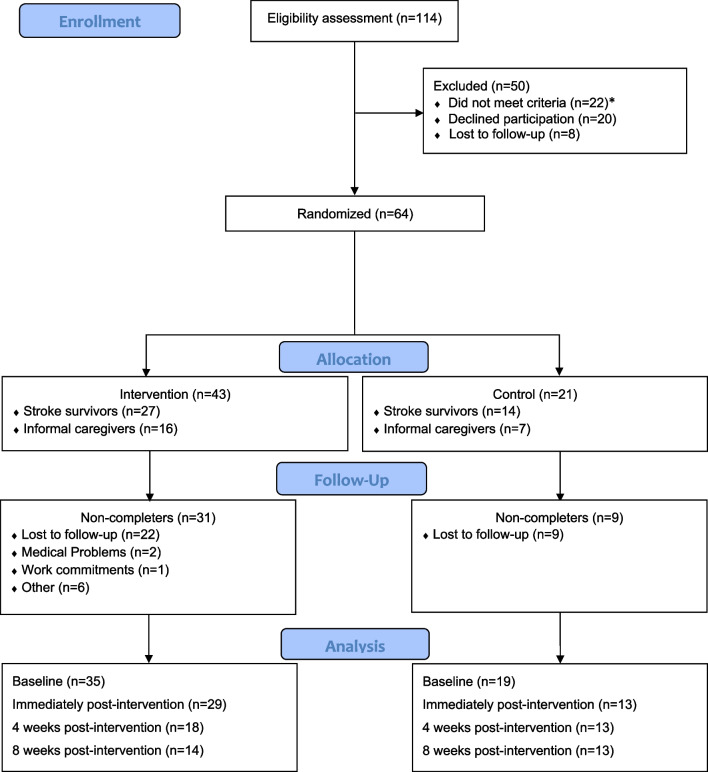
Flow diagram. *3 stroke survivors were found ineligible after randomization to the intervention and excluded prior to intervention initiation

Recruitment began in September 2017 and ended in November 2018, and data collection was completed in January 2019. Of the 114 adults assessed for eligibility, seventy-one individuals enrolled and 64 were randomized (Fig. 1). No study related harms were reported. The intervention group consisted of 11 SS-IC dyads and 16 SS and 5 IC who participated without a counterpart. The control group consisted of 5 SS-IC dyads and 9 SS and 2 IC who participated without a counterpart. As outlined in Table 1, the mean days since stroke was 105.86 (standard deviation (SD), ± 101.07), and the median was 56. Among 33 SS, mean baseline CES-D scores were 18 ± 13 SD. Among 20 IC, mean CES-D scores were 11 (± 9 SD) indicating minimal risk for depression. Forty-four participants (n = 29 SS, 71%; n = 15 IC, 65%) were administered DIGS [22] questionnaires. Among the SS, major depressive disorder, 8 (28%); and substance/medication-induced depressive disorder, 1 (3%) were determined. Among the IC, major depressive disorder, 11 (25%); substance/medication-induced depressive disorder, 2 (5%); and dysthymia, 1 (2%) were determined.

**Table 1:** Sample characteristics

	SS n = 41	IC n = 23
	n (%)^a^	n (%)
Male sex	13 (32)	4 (17)
Age, mean years (± SD)	57 (± 14)^b^	52 (± 15)
Time since stroke, mean days (± SD)	106 (± 101)	–
Race		
Non-Hispanic black	17 (49)	9 (43)
Non-Hispanic white	12 (34)	6 (29)
Hispanic	6 (17)	5 (24)
Highest level education		
High school or equivalent	10 (29)	4 (19)
Some college/vocational	14 (40)	12 (57)
College degree	11 (31)	5 (24)
Income		
≤ $49,999	20 (59)	10 (48)
$50,000–$99,999	6 (18)	6 (29)
≥ $100,000	8 (24)	5 (24)
Stroke type		
Ischemic	26 (63)	–
Hemorrhagic	5 (12)	–
Transient ischemic attack	10 (24)	–
Antidepressant		
Yes	17 (42)	0 (0)
No	24 (59)	15 (65)
Anxiolytic		
Yes	11 (27)	0 (0)
No	30 (73)	15 (65)
Mean baseline score (± SD)		
CES-D	18 (± 13) n = 33	11 (± 9) n = 20
STAI-S	41 (± 14) n = 34	37 (± 14) n = 20
STAI-T	40 (± 14) n = 34	38 (± 13) n = 20
BPI-sf severity	4 (± 3) n = 29	3 (± 2) n = 20
BPI-sf interference	3 (± 3) n = 31	2 (± 3) n = 20

^a^Percentages may not total 100% due to rounding or missing values

^b^Age at time of stroke

### Feasibility

The enrollment rate was 77%. Of the 71 enrollees, 26 (37%) completed the study. However, 42 (66%) participants completed baseline and immediate post-intervention visits. Of the SS non-completers, 9 (50%) were men, the mean age was 57 ± 13, and 10 (56%) were non-Hispanic Black, 5 (28%) non-Hispanic White, and 3 (17%) Hispanic ethnicity. Of the IC non-completers, 3 (22%) were men, the mean age was 50 ± 17, and 5 (36%) were non-Hispanic Black, 3 (21%) non-Hispanic White, and 5 (36%) Hispanic ethnicity.

The mean questionnaire completion rate was 95%, with rates of 96% for participants in the intervention group and 93% for participants in the control group. Fifty-four (84%) participants completed questionnaires at baseline compared to 42 (66%) participants at week 4, 31 (48%) participants at week 8, and 27 (42%) participants at week 12. In total, 12 SS requested, at least once, to complete the questionnaires via pen and paper versus electronic questionnaires compared to four IC. At baseline, 67 (100%) participants provided a blood sample, including the three participants who were deemed ineligible after enrollment. Immediately after intervention completion, 32 (50%) of the 64 participants provided a blood sample, compared to 21 (33%) participants 4 and 8 weeks after intervention completion.

The median meditation group session attendance rate was 75.0%, and the mean attendance rate was 55%. SS mean attendance rate was 59% (median of 76%) and 48% (median of 50%) in IC. Thirty-one (72%) participants attended at least one session. Among these 31 participants, 71% attended 3 or 4 sessions, and 94% attended at least 2 sessions. Both participants in 7 of the 11 dyads (64% of dyads) attended either 3 or 4 sessions. Of the 16 SS enrolled as individuals, 81% attended at least 2 sessions. In contrast, among the 5 IC enrolled as individuals, 40% did not attend any sessions, and the remaining 60% attended 1 or 2 sessions.

Of the 43 participants in the intervention group, 28 (65%) completed at least one daily meditation practice questionnaire. Among the SS, 70% submitted at least one practice questionnaire, compared to 56% of the IC. The mean proportion of questionnaires indicating participants had meditated was 96%. Practice questionnaire responses indicated meditation frequency ranged from three times a week to several times daily. Of the 19 participants who completed at least one questionnaire after the intervention phase, 17 (89%) meditated. See Additional File 1 for the qualitative data analyses.

### Efficacy

#### Within group

Among SS, paired t-tests indicated non-significant reductions in CES-D scores from baseline to week 4 (mean difference = − 3.3000 [95% confidence interval (CI) − 8.4540, 1.8540] *p* = 0.1960), baseline to week 8 (mean difference = − 5.0000 [95% CI − 12.7201, 2.7201] *p* = 0.1818), and baseline to week 12 (mean difference = − 4.6000 [95% CI − 14.9621, 5.7621] *p* = 0.3415). Although evidence of a difference was not shown by paired *t* tests, SS had reductions in STAI-State scores from baseline to 4 weeks (mean difference = − 5.8500 [95% CI − 11.7236, 0.0236] *p* = 0.0508), baseline to 8 weeks (mean difference = − 6.9231 [95% CI − 14.8116, 0.9655] *p* = 0.08), and baseline to 12 weeks (mean difference = − 8.2000 [95% CI − 17.1144, 0.7144] *p* = 0.0672). Paired *t* tests for SS indicated a reduction from baseline to week 8 in BPI-sf Severity scores (mean difference = − 1.4792 [95% CI − 2.7561, − 0.2022] *p* = 0.0270).

Among IC, no significant changes in CES-D scores were observed from baseline to week 4 (mean difference = − 2.8750 [95% CI − 10.8993, 6.1493] *p* = 0.4249), baseline to week 8 (mean difference = − 8.000 [95% CI − 29.6235, 13.6235] *p* = 0.3239), and baseline to week 12 (mean difference = − 0.6667 [95% CI − 20.7457, 19.4124] *p* = 0.8995). Repeated measures analysis with linear mixed models indicated a statistically significant reduction in STAI-Trait scores (F (_3,16.2)_ = 3.28, *p* = 0.0479). A paired t-test showed a significant reduction from baseline to week 4 in STAI-Trait scores (mean difference = − 9.1250 [95% CI − 16.8060, − 1.4440], *p* = 0.0262). Although evidence of a difference was not shown by paired *t* test, STAI-State scores were reduced at 4 weeks (mean difference = − 11.7500 [95% CI − 24.419, 0.919] *p* = 0.0644). Paired *t* test results were non-significant for BPI-sf Intensity from baseline to week 4 (mean difference 0.2679 [95% CI − 3.5226, 4.0583] *p* = 0.8720), baseline to week 8 (mean difference − 0.0714 [95% CI − 6.4701, 6.3272] *p* = 0.9739), and baseline to week 12 (mean difference 0.6095 [95% CI − 19.1279, 20.3670] *p* = 0.9066) and for BPI-sf Severity from baseline to week 4 (mean difference − 0.8125 [95% CI − 3.6382, 2.0132] *p* = 0.5184), baseline to week 8 (mean difference − 2.00 [95% CI − 5.4527, 1.4527] = 0.1625), and baseline to week 12 (− 2.3333 [95% CI − 16.4966, 11.8330] *p* = 0.5520).

#### Between groups

Repeated measures analysis with linear mixed models indicated no significant differences for the interaction of group x time for any outcome variables.

## Discussion

Our longitudinal investigation was different from the few known meditation studies in stroke populations [29, 30] in that the relatively young and racially diverse sample included also IC as active participants. Collectively, our findings suggest additional strategies are needed to optimize the feasibility of the meditation intervention before executing a large-scale RCT powered for efficacy testing. The main challenges to feasibility were recruitment of IC and dyads and participant retention and intervention adherence.

In addition to the lengthy recruitment period, the number of individual participants was double the number of individuals participating in a dyad and fewer IC than SS enrolled in the study. To maintain the enrollment rate, the original inclusion criteria were revised to allow for individual SS and IC to participate aside from dyads. Known barriers associated with dyad enrollment include a lack of participation readiness by both members of the dyad [31]. On average stroke survivor participants experienced their stroke event approximately 3 months prior to study enrollment. Willingness to participate may increase over time, as SS and IC often struggle with the suddenness of the stroke and related dependency [9]. IC preparedness and the demands of the caring role are known barriers to IC participation in stroke dyad interventions [32]. Future studies might consider re-contacting potential dyads to reassess their readiness [31] and personalized interventions (e.g., enrolling IC alone rather than in dyads, individual rather than group meditation, and alternative modes of intervention delivery including online [11]) that consider the needs of the IC. Additional strategies (e.g., compensation, community partnerships, and digital recruitment activities) for community-based recruitment might also be considered for future studies [33].

Sixty-six percent of the participants were retained compared to the pre-set 80% retention criteria at the 8-week post-intervention visit. However, 83% of the participants assigned to the meditation group were retained through the 4-week intervention. The additional 4- and 8-week post-intervention follow-up visits were incorporated to evaluate the feasibility of evaluating sustained meditation efficacy beyond the immediate structured 4-week meditation period. Most non-completers were lost to follow-up, defined as incomplete ascertainment of depressive symptom data through the 8-week post-intervention visit. We routinely (e.g., once a week for 3 weeks) attempted to contact participants who did not attend their scheduled visits. Repeated attempts at re-contact were mostly unsuccessful. Due to budgetary and timeline restraints of our feasibility study, we were unable to implement a comprehensive retention plan (e.g., trial innovation network toolbox) [34]. Questionnaire burden (e.g., length of each questionnaire and difficulty in providing the requested data) blood sample collection (e.g., number and timing of required post-intervention blood draws) may be considered in future studies. Important to note, participants identified distance and lack of transportation as predominate barriers to participation. Pre-randomization screening of demographic and other factors (e.g., transportation) may facilitate the identification of those at greatest risk for non-completion and retention strategies tailored to this group. Establishing a realistic budget for participant travel and parking reimbursement is necessary. Most SS non-completers were non-Hispanic Black men, whereas IC non-completers tended to be Hispanic females. Culturally sensitive meditation interventions (e.g., employing mind–body experts of the same race and ethnicity) may support the retention of racially and ethnically diverse participants [35].

The DIGS questionnaire was administered during a telephone interview at baseline, was approximately 1 h, and may have influenced rates of withdrawal or loss to follow-up. The questionnaire was incorporated to evaluate the feasibility of including a formal diagnostic interview for major mood and other psychiatric disorders and conditions. The use of a shorter diagnostic measure that exclusively evaluates depression may be considered in future studies. Since few participants routinely completed daily meditation practice questionnaires, it is unclear if participants established daily meditation practice. Qualitative reports from the participants assigned to the intervention group indicated that the daily email reminders to complete the meditation practice questionnaire were confusing. Furthermore, some participants indicated difficulties *using a computer* (e.g., physical effects of stroke and lack of technical knowledge). Methodological considerations in the future to promote completion of a daily electronic questionnaire might include pre-intervention screening for potential technology-related challenges, ongoing or additional technical supports (e.g., alternative hardware options), and tailoring reminders and access based on individual preference (e.g., short message services and interactive voice response) [36].

Intervention adherence was lower (mean session attendance rate of 55%) than our established criteria for adherence (75% or greater session attendance). Almost all intervention participants attended at least two group sessions. A meta-analysis of the effects of meditation on depression found that group meditation conferred a greater reduction in depressive symptoms than individual meditation, and a greater duration of unstructured meditation demonstrated a greater reduction in depressive symptoms [37]. Participants were more likely to attend at least three sessions when they enrolled as part of a dyad. These findings are not surprising considering the amount of caregiving being delivered by informal caregivers in the post-stroke recovery period [38]. Future studies should consider emphasizing the potential benefits to both members of the dyad when IC are active participants, offering online modes of intervention delivery (e.g., video conference and virtual environments) and technologies to support ongoing participation, SS and IC engagement in trial design and implementation, and the need to investigate dose–effect associations between meditation and benefits to optimize meditation interventions in the stroke population.

The primary objective was to determine the feasibility of a 4-week meditation RCT in the stroke population. Inferences as to the effects of the meditation intervention on depression symptom severity, anxiety, and pain should be made with caution [39]. Furthermore, participants without symptoms of depression, anxiety, or pain or with subclinical symptoms were included in the analyses; therefore, improvements in symptoms from the meditation intervention were less likely. Meditation may be more effective for those with greater depression and anxiety symptomatology or higher levels of pain. These results, however, are hypothesis stimulating and warrant further exploration with a larger sample.

### Limitations

Participants were mostly female with some college or training and recruited from a single clinic, limiting the generalizability. Furthermore, stroke survivors with severe cognitive deficits or aphasia were excluded due to our informed consent process. Strategies for inclusion (e.g., aphasia-friendly research documents) may be considered in future studies [40, 41]. Selection bias may have resulted from the use of convenience sampling and performance bias due to lack of blinding measures. While the application of various blinding techniques in trials of behavioral interventions is sometimes impractical or infeasible, strategies for blinding should be considered in a future study. Limitations related to the qualitative data were the lack of formal semi-structured interviews, the size and number of the unstructured group interviews, and the involvement of the PI, research staff, and senior meditation teacher. Structured group interviews facilitated by an impartial individual and using a semi-structured interview guide with participants assigned to the intervention and control groups, as well as those who withdrew from the study, would have allowed for greater depth and breadth of the data obtained and decreased bias. Limitations related to our quantitative data include threats to validity and reliability of the self-report measurements. For future meditation efficacy trials, methods other than self-report (e.g., biological measures of meditation efficacy) may be considered along with self-reported measures of participants’ subjective experience of their own emotions and behaviors. While the task structure was standardized between intervention and control conditions, differences in the expected time commitment and mode of delivery (e.g., group versus individual) persisted. Future RCTs of group-based meditation interventions might consider group-based control arms to address the possible group interaction effects that may interfere with the evaluation of treatment effects.

## Conclusions

Despite high enrollment rates, the lengthy recruitment period, and low retention and adherence rates suggest methodological changes are needed before intervention efficacy testing in a larger RCT. Consideration needs to be given to the feasibility of an in-person, multiple-visit meditation intervention. Internet-based and mobile technologies have potential to overcome geographical and transportation barriers and support sustained participation in meditation interventions after stroke. However, barriers and best practices of racial and ethnic minority and IC’s involvement must also be considered.

## Supplementary Information

**Additional file 1**. Qualitative data analyses included grouping the qualitative data (e.g., unstructured group interviews and field notes) and the reasons for declining stud participation into categories.

## Data Availability

Anonymized data are available upon reasonable request and with the permission of the UTHealth Houston Institutional Review Board. Requests should be sent to Dr. Jennifer Beauchamp at jennifer.e.beauchamp@uth.tmc.edu.

## Abbreviations

SS: Stroke survivor(s)
IC: Informal caregiver(s)
RCT: Randomized controlled trial
REDCap: Research electronic data capture
CES-D: Center for Epidemiologic Studies Depression
STAI: State-Trait Anxiety Inventory for Adults
BPI-sf: Brief Pain Inventory-Short Form
SD: Standard deviation
CI: Confidence interval
