# Supplementary material for: Feasibility of a meditation intervention for stroke survivors and informal caregivers: a randomized controlled trial
Source: BMC Psychol. 2023 Jan 12;11:9. doi: 10.1186/s40359-022-01031-z (PMC9838004; doi:10.1186/s40359-022-01031-z)
Supplement: Supplementary file 1 — Additional file 1. Qualitative data analyses included grouping the qualitative data (e.g., unstructured group interviews and field notes) and the reasons for declining stud participation into categories. [file 40359_2022_1031_MOESM1_ESM.docx]

**ADDITIONAL FILES**

Additional File 1: Qualitative Data Analyses; Qualitative data analyses included grouping the qualitative data (e.g., unstructured group interviews and field notes) and the reasons for declining study participation into categories.

**Supplementary Material. Qualitative Data Analyses.**

Unstructured group interviews were completed with 19 (n = 12 SS and n = 7 IC) of the 43 participants from the nine intervention cohorts. Field notes were documented on 47 participants from the intervention and control groups. Qualitative data indicated distance and transportation were primary barriers to participation; however, the majority voiced favorable responses (e.g., increased feelings of relaxation and improvements in sleep) to meditation. One participant reported, “When I would I have an anxiety attack, I would start the breathing and the relaxation, and it would calm me down.” Other participants stated, “I began to feel connected to the universe,” and “I felt more grounded.” Internalization of meditation teachings was evidenced by reports of meditating to alleviate “monkey brain” or “chatter mind,” as well as the use of the “STOP” technique to manage responses to stressful situations. Some participants reported instances in which meditation improved physical symptoms (e.g., reductions in the severity and frequency of headaches). Some participants indicated that they planned to continue meditation after study completion. A few participants reported challenges, such as finding it “very hard to relax” or finding meditation unhelpful “when I wake up at 3:00 a.m.”

Comments gathered from the interview data and field notes emphasized issues with email communication and technology. Some participants reported difficulties using a computer, owing to either the physical effects of the stroke on their upper extremities or a lack of technological knowledge. Some participants reported physical barriers (e.g., accessing the parking garage and the building) to meditation group session attendance. Others reported being confused by the day-to-day email reminders to complete the meditation practice questionnaires, resulting in difficulty locating other study emails.

Reasons for non-participation included distance or lack of transportation (n=10), time commitment (n=4), lack of interest (n=2), blood sample collections (n=1), and medical problems (n=1).
